# Adolescent Suicide: A Global Meta-Analysis of Genetic, Biological, Neuroimaging and Sociocultural Risk Factors

**DOI:** 10.1192/bjo.2026.11245

**Published:** 2026-06-30

**Authors:** Baidyanath Ghosh Dastidar, Prathama Guha

**Affiliations:** 1 Kolkata, Kolkata, India; 2 Calcutta National Medical College, Kolkata, India

## Abstract

**Aims:**

Adolescent suicide is a leading cause of mortality worldwide, yet prediction remains limited by symptom-based clinical assessment. This study aimed to synthesise genetic, biological, neuroimaging and sociocultural evidence to develop a mechanistically informed framework for adolescent suicide risk, moving beyond single-domain or correlational models.

**Methods:**

We conducted a PRISMA-compliant systematic review and meta-analysis registered with PROSPERO (CRD420251020839). Searches of PubMed, EMBASE, PsycINFO, CENTRAL, Scopus and Web of Science identified studies published between 2005 and 2025 involving adolescents aged 10–19 years reporting suicidal ideation, suicide attempts or death by suicide. Eligible studies contributed data from at least one domain: genome-wide association studies or polygenic risk scores, neuroimaging, inflammatory or neurotrophic biomarkers, sociocultural risk factors, or treatment outcomes related to lithium, sodium valproate or electroconvulsive therapy (ECT). Random-effects meta-analysis generated pooled effect sizes, with heterogeneity quantified using I². Machine-learning analyses used harmonised study-level estimates only, employing XGBoost with nested cross-validation and SHAP interpretation. Mendelian randomisation was applied to explore genetically informed pathways.

**Results:**

A total of 140 studies comprising 1,526,000 adolescents from 80 countries were included. The pooled effect size for suicide risk across domains was d=0.71 (95% CI 0.58–0.83; I²=78%). Significant predictors included polygenic risk for major depressive disorder (r=0.21), elevated interleukin-6 (r=0.38) and C-reactive protein (r=0.32), ventromedial prefrontal cortex hypoactivity (r=−0.31), childhood trauma (r=0.42) and sexual and gender minority stress (r=0.44) (all p<0.001). An integrative Bio-Psycho-Social Suicide Risk Index achieved an internally validated area under the curve of 0.93, interpreted as hypothesis-generating. Mendelian randomisation supported genetically informed pathways linking liability to inflammation and neural dysfunction. Secondary exploratory analyses suggested associations between lithium and reduced inflammatory markers, sodium valproate and improved inhibitory neurotransmission, and ECT and increased hippocampal volume.

**Conclusion:**

Adolescent suicide reflects interacting genetic, neurobiological and sociocultural processes. This global meta-analysis provides the most comprehensive mechanistic synthesis to date and proposes a biologically informed framework to complement clinical judgement. Prospective external validation is required before clinical implementation.

